# At the end of life, humanity still matters

**DOI:** 10.1017/S1478951526101904

**Published:** 2026-02-24

**Authors:** Daliya Rizvi, Karen Jooste

**Affiliations:** 1 Department of Biology, Duke University, Durham, NC, USA; 2 Department of Pediatrics, Duke University, Durham, NC, USA; 3 Department of Medicine, Palliative Care, Duke University, Durham, NC, USA

Recently, I found myself at a prestigious research conference watching a panel of cardiologists attempting to outsmart artificial intelligence (AI). Both the physicians and the AI platform were repeatedly presented with a sample case and asked to make the appropriate diagnosis. And in every case, AI was able to diagnose the patient more quickly and seemingly, more accurately. The final verdict, rather depressingly, was “AI: 10, Humanity: 0.”

As AI assumes a more prominent role in society, clinicians and patients alike have begun relying on its convenience and projections of confidence and authority to aid in diagnoses, inform medical decisions, and help determine optimal courses of action. It’s easy to see why – type a rudimentary amount of health information into ChatGPT, and you’re given a list of potential conditions you may be afflicted with, step-by-step advice on how to combat your symptoms, and best of all, the reassurance that you were looking for all along. Our healthcare system is full of barriers that make it difficult to get the answers we are seeking; AI can give them to us with the stroke of a few keys. Conversations with doctors are often limited to 15-min windows; AI has all day to listen to us. With insurance premium costs on the rise, increasing drug prices, and the fact that a simple physical examination can cost hundreds for someone without insurance, many are turning to generative AI to take on the role that a primary care physician once held in their lives.

This is problematic for several reasons. While AI can analyze data and identify patterns, it cannot fully grasp a patient’s personal circumstances and values. Medical decisions often require balancing risks, patient preferences, and ethical considerations – nuances that are inherently subjective and complex. Human beings come from a variety of racial, religious, social, and cultural backgrounds that shape their experiences in the healthcare system. AI may recognize patterns related to the social determinants of health, but it lacks the insight needed to grasp the complex social realities, histories, and inequities that give those patterns real meaning. Furthermore, medicine frequently involves uncertainty. Physicians must make decisions with incomplete, ambiguous, or contradictory information. AI depends on existing data patterns, which makes it vulnerable when confronted with rare conditions, atypical presentations, or emerging diseases. Human clinicians, in contrast, can integrate intuition, experience, and critical thinking into their practice to navigate these situations.

Perhaps the most troubling aspect of all of this, however, is the use of AI in healthcare at the cost of human empathy. Some things are impossible to properly describe with words. The comfort we get from sharing a smile; the way that even the heaviest burdens can become lighter when we laugh with someone we love. The compassion that comes with the humanity of healthcare that cannot be replicated by AI, no matter how advanced it becomes. The American Psychological Association has reported an alarming increase in the number of people who turn to AI for emotional support. While such support is a foundation of overall well-being, overreliance on technology for relationships that should be formed with other human beings has led to tragic outcomes, including the mismanagement of suicidal and self-harming behaviors.

In a field like Palliative Medicine in particular, the human touch is a crucial aspect of facilitating a graceful transition from life to death. Unlike other medical specialties, where success is often defined by measurable outcomes, such as tumor shrinkage, restored mobility, or extended survival, Palliative Care operates in a realm where the goals are more nuanced and profoundly human. It is rife with clinical dilemmas that are not puzzles to be solved but dissonant human stories in need of a resolution. In these circumstances, the most effective interventions are rarely purely medical. Instead, they revolve around communication, empathy, and the ability to sit with discomfort – both the patient’s and one’s own. Palliative Care specialists must learn to listen not only for symptoms but for values and fears, for the unspoken concerns that shape how patients and families understand dying. They must navigate uncertainty with sensitivity, recognizing that their presence can ease burdens that medicine cannot cure.

I recently had the privilege of reading a letter written by a patient treated at the Hock Family Pavilion inpatient hospice facility at the Duke University Medical Center. E.S. was only 47 years old when he succumbed to a battle with colon cancer. The letter, which was shared with permission from his wife and read after his death, was addressed to the hospice nurses and staff he encountered in the facility.

“Your dedication to my comfort and well-being, the gentleness with which you treated me, and the respect you showed for my dignity […] were more than just professional duties; they were acts of genuine love and kindness,” he wrote. “Your work is not just a job; it is a calling, and you answer it with compassion […]”

The humanity of medicine becomes especially vital as patients confront the end of life, a time characterized by profound vulnerability. A gentle tone of voice, a careful explanation, a few unhurried moments at the bedside – these seemingly small gestures can restore dignity and help patients feel seen when so much else is being lost. Families, too, rely on clinicians not just for guidance but for reassurance that their loved one is being cared for as a whole person, not an inevitable outcome. True care goes beyond performing tasks correctly; it requires respect for dignity and acts of genuine love – qualities that are deeply human and cannot be programmed. The profound comfort the patient describes comes from emotional presence, moral sensitivity, and being fully attuned to another person’s needs, fears, and vulnerabilities. AI can provide guidance, reminders, or logistical support, but it cannot authentically feel, empathize, or offer the warmth of human touch and presence. Palliative Care is not just about service; it is about shared humanity, and it is in this human connection that true healing and comfort are found.

While AI offers tools for diagnosis, data analysis, and logistical support, it cannot replicate the profoundly human elements that lie at the heart of healthcare. Erik didn’t mention the top-quality medical treatment he received at Duke in his letter. He said nothing about his treatment regimen or the state-of-the-art equipment and technology. What truly stuck with him were the empathy, compassion, and moral discernment displayed by his providers. Practicing medicine is not only about identifying patterns or applying protocols. It requires the ability to listen deeply, to interpret unspoken fears, to offer reassurance through presence, tone, and touch, and to honor the dignity of each person as a unique individual. In Palliative Care, these qualities are not optional but essential, because they shape the ways in which patients experience their final moments. In primary care and routine medical encounters, the human elements of kindness, patience, and emotional attunement create trust, foster healing, and provide comfort in ways that no algorithm can measure or replicate. AI can guide decisions and enhance efficiency, but the act of caring – the relational, moral, and emotional labor that defines medicine – remains a distinctly human calling. Ultimately, the value of healthcare, particularly end-of-life care, lies not merely in outcomes or speed but in the human connections that bring dignity, reprieve from grief, and meaning to patients’ lives when they are most vulnerable. After all, grief is often an expression of love. And what could be more profoundly human than love?

